# Nutritional quality of diet and academic performance in Chilean students

**DOI:** 10.2471/BLT.15.161315

**Published:** 2016-02-03

**Authors:** Paulina Correa-Burrows, Raquel Burrows, Estela Blanco, Marcela Reyes, Sheila Gahagan

**Affiliations:** a Institute of Nutrition and Food Technology, University of Chile, Avda. El Líbano 5524, Macul, CP 7830490, Santiago de Chile, Chile.; b Division of Child Development and Community Health, University of California San Diego, San Diego, United States of America.

## Abstract

**Objective:**

To explore associations between the nutritional quality of diet at age 16 years and academic performance in students from Santiago, Chile.

**Methods:**

We assessed the nutritional quality of diet, using a validated food frequency questionnaire, in 395 students aged 16.8 ± 0.5 years. Depending on the amount of saturated fat, fibre, sugar and salt in the foods, diet was categorized as unhealthy, fair or healthy. Academic performance was assessed using high school grade-point average (GPA) and tests for college admission in language and mathematics. Academic results on or above the 75th percentile in our sample were considered good academic performance. We tested associations between nutritional quality of diet and good academic performance using logistic regression models. We considered sociodemographic, educational and body-mass index (BMI) factors as potential confounders.

**Findings:**

After controlling for potential confounding factors, an unhealthy diet at age 16 years was associated with reduced academic performance. Compared to participants with healthy diets, those with unhealthy diets were significantly less likely to perform well based on language tests (odds ratio, OR: 0.42; 95% confidence interval, CI: 0.18–0.98) mathematics tests (OR: 0.35; 95% CI: 0.15–0.82) or GPA (OR: 0.22; 95% CI: 0.09–0.56).

**Conclusion:**

In our sample, excessive consumption of energy-dense, low-fibre, high-fat foods at age 16 years was associated with reduced academic performance.

## Résumé

**Objectif:**

Étudier les liens entre la qualité nutritionnelle de l'alimentation à 16 ans et les résultats scolaires des lycéens de Santiago, au Chili.

Nous avons estimé la qualité nutritionnelle de l'alimentation de 395 lycéens âgés de 16,8 ans (± 0,5 an) à l'aide d'un questionnaire validé permettant de déterminer la fréquence de consommation de produits alimentaires. En fonction de la quantité de graisses saturées, de fibres, de sucre et de sel contenue dans ces aliments, leur alimentation a été catégorisée comme mauvaise, équilibrée ou saine. Les résultats scolaires ont été évalués à partir de la moyenne générale et des examens d'admission à l'université en langues et en mathématiques. Les résultats supérieurs ou égaux au 75e centile de notre échantillon ont été considérés comme bons résultats scolaires. Nous avons testé les liens entre qualité nutritionnelle de l'alimentation et bons résultats scolaires à l'aide de modèles de régression logistique. Nous avons considéré les facteurs socio-démographiques, éducatifs et liés à l’indice de masse corporelle (IMC) comme facteurs de confusion potentiels.

Après contrôle des facteurs de confusion potentiels, il est apparu qu’une mauvaise alimentation à l'âge de 16 ans était liée à de moins bons résultats scolaires. Comparés aux participants ayant une alimentation saine, ceux ayant une mauvaise alimentation avaient sensiblement moins de chances d'obtenir de bons résultats aux examens de langues (rapport des cotes, RC: 0,42; intervalle de confiance (IC) de 95%: 0,18–0,98), aux examens de mathématiques (RC: 0,35; IC 95%: 0,15–0,82) ou en termes de moyenne générale (RC: 0,22; IC 95%: 0,09-0,56).

Dans notre échantillon, la consommation excessive de produits à forte teneur calorique, pauvres en fibres et riches en matière grasse à l'âge de 16 ans était liée à de moins bons résultats scolaires.

## Resumen

**Objetivo:**

Explorar las relaciones entre la calidad nutricional de la dieta de estudiantes de 16 años de Santiago (Chile) y su rendimiento académico.

**Métodos:**

Se evaluó la calidad nutricional de la dieta, mediante un cuestionario validado sobre la frecuencia de consumo de alimentos, de 395 estudiantes de 16,8 ± 0,5 años. Según la cantidad de grasas saturadas, fibra, azúcar y sal en los alimentos, se ha categorizado la dieta como insalubre, razonable o saludable. Se evaluó el rendimiento académico utilizando el promedio de las calificaciones (GPA, por sus siglas en inglés) del instituto y pruebas de idiomas y matemáticas realizadas para la admisión en los centros. Los resultados académicos iguales o superiores al 75% en nuestra muestra se consideraron como un buen rendimiento académico. Se probaron las relaciones entre la calidad nutricional de la dieta y el buen rendimiento académico mediante modelos de regresión logística. Se tuvieron en cuenta factores sociodemográficos, educativos del índice de masa corporal (IMC) como posibles factores de confusión.

**Resultados:**

Tras controlar los posibles factores de confusión, se relacionó una dieta insalubre en estudiantes de 16 años con un peor rendimiento académico. En comparación con los participantes con dietas saludables, los que seguían dietas insalubres tenían más posibilidades de obtener peores resultados en las pruebas de idiomas (cociente de posibilidades, CP, de 0,42; intervalo de confianza, IC, del 95%: 0,18–0,98) pruebas de matemáticas (CP de 0,35; IC del 95%: 0,15–0,82) o GPA (CP de 0,22; IC del 95%: 0,09–0,56).

**Conclusión:**

En nuestra muestra, el consumo excesivo de alimentos altamente energéticos, bajos en fibra y con elevadas cantidades de grasa en estudiantes de 16 años está relacionado con un peor rendimiento académico.

## ملخص

**الغرض:**

استكشاف علاقات الاقتران ما بين القيمة الغذائية للنظام الغذائي المتبع في سن 16 عامًا والأداء الأكاديمي لدى الطلاب في سانتياغو بشيلي.

**الطريقة:**

قمنا بتقييم القيمة الغذائية للنظام الغذائي باستخدام استبيان تم توثيق مصداقيته لمعدل تكرار تناول الطعام، على 395 طالبًا تتراوح أعمارهم بين 16.8 ± 0.5 أعوام. واستنادًا إلى كمية الدهون المشبعة والألياف والسكر والملح في الأطعمة، تم تصنيف النظام الغذائي بكونه غير صحي أو معتدل أو صحي. تم تقييم الأداء الأكاديمي باستخدام متوسط درجات الشهادة الثانوية (GPA) واختبارات القبول في الكليات في مواد اللغات والرياضيات. إن الطلاب الحاصلين على نتائج أكاديمية تبلغ 75 أو أعلى في مقياس النسبة المئوية في عينتنا تم اعتبارهم من ذوي الأداء الأكاديمي الجيد. وقمنا باختبار علاقات الاقتران بين القيمة الغذائية للنظام الغذائي والأداء الأكاديمي الجيد، وذلك باستخدام نماذج التحوف اللوجيستي. كما قمنا بمراعاة عوامل الخطر الاجتماعية والديموغرافية والتعليمية ومؤشر كتلة الجسم (BMI) باعتبارها متغيرات مربكة محتملة.

**النتائج:**

بعد التحكم في العوامل المربكة المحتملة، اقترن النظام الغذائي غير الصحي في سن 16 عامًا بتدني الأداء الأكاديمي. وبالمقارنة مع المشاركين الذين يتبعون أنظمة غذائية صحية، وجدنا أن المشاركين الذين يتبعون أنظمة غذائية غير صحية هم أقل عرضة للقيام بأداء جيد بناءً على اختبارات اللغات (نسبة الاحتمال: 0.42؛ بنسبة أرجحية مقدارها ‏‏95%: 0.18–0.98) أو اختبارات الرياضيات (نسبة الاحتمال: 0.35؛ ونسبة أرجحية مقدارها 95‏‏%: 0.15–0.82) أو متوسط درجات الشهادة الثانوية (نسبة الاحتمال: 0.22؛ ونسبة أرجحية مقدارها 95‏‏%: 0.09–0.56).

**الاستنتاج:**

وجدنا في العينة الخاصة بنا أن التناول المفرط للأطعمة المكونة من عناصر مليئة بالطاقة ومنخفضة الألياف وعالية الدهون في سن 16 عامًا قد اقترن بتدني الأداء الأكاديمي.

## 摘要

**目的:**

旨在探索智利圣地亚哥 16 岁学生摄入的营养状况和其学习成绩之间的联系。

**方法:**

我们在 395 名 16.8 ± 0.5 岁的学生中展开有效的食物频率问卷调查，评估其饮食的营养状况。基于食物中饱和脂肪、纤维、糖和盐的含量，饮食被分为不健康、一般和健康三类。通过高等学校平均积分点 (GPA) 和大学入学语言和数学测试评估学习成绩。学习成绩为我们样例的 75% 或以上，则被认为学习成绩优良。我们利用逻辑回归模型检测了饮食营养状况和学习成绩优良之间的联系。我们认为社会人口、教育和体重指数（BMI）因子为潜在的混淆因素。

**结果:**

控制潜在的混淆因素之后，16 岁饮食不健康则会降低其学习成绩。相对于饮食健康的参与人员，饮食不健康的人则明显不太可能表现良好，语言测试（优势率，OR：0.42；95% 置信区间，CI：0.18–0.98）数学测试（OR：0.35；95% CI：0.15-0.82）或平均积分点（OR：0.22；95% CI：0.09–0.56）。

**结论:**

在我们的样例中，16 岁过量食入高热量、低纤维、高脂肪食物，会导致学习成绩下降。

## Резюме

**Цель:**

Изучить связь между пищевой ценностью рациона и успеваемостью 16-летних учащихся г. Сантьяго, Чили.

**Методы:**

С помощью утвержденного опросника частоты потребления пищевых продуктов была проведена оценка пищевой ценности рациона у 395 учащихся, возраст которых составлял 16,8 ± 0,5 года. В зависимости от количества насыщенного жира, клетчатки, сахара и соли в пищевых продуктах питание было определено как нездоровое, удовлетворительное или здоровое. Для оценки успеваемости были использованы: средний академический балл (GPA) в старшей школе и вступительные тесты в колледж по языку и математике. Результаты, равные процентилю 75 или превышающие его в данной выборке, трактовались как хорошая успеваемость. Связи между пищевой ценностью рациона и хорошей успеваемостью были проверены с помощью моделей логистической регрессии. В качестве потенциальных, искажающих результаты факторов были приняты социально-демографические, образовательные факторы, а также индекс массы тела.

**Результаты:**

После внесения поправок на факторы, способные искажать результаты, была установлена связь между нездоровым питанием в возрасте 16 лет и пониженной успеваемостью. Успеваемость лиц с нездоровым рационом, в отличие от участников, практикующих здоровое питание, с меньшей вероятностью могла быть охарактеризована как хорошая на основании их тестов по языку (отношение шансов, ОШ: 0,42; 95% доверительный интервал, ДИ: 0,18–0,98), тестов по математике (ОШ: 0,35; 95% ДИ: 0,15–0,82) или GPA (ОШ: 0,22; 95% ДИ: 0,09–0,56).

**Вывод:**

В данной выборке была установлена связь между сниженной успеваемостью и чрезмерным потреблением 16-летними учащимися высококалорийных продуктов с низким содержанием клетчатки и высоким содержанием жиров.

## Introduction

Health-related behaviours may play a role in the ability to learn. Although critical stages of brain maturation occur early in life, the development of certain structures and higher cognitive functions (e.g. abstract thinking, deductive reasoning and problem solving) takes place in adolescence and continues during early adulthood.^1^^–^^3^ Brain development is strongly influenced by environmental factors, with nutrition playing a pivotal role.^4^ Whereas the effect of nutritional deficits on cognition are well known, the impact of overnutrition became the focus of research in the past decade.^2^^,^^5^^,^^6^

In the United Kingdom of Great Britain and Northern Ireland, diet during infancy was associated with intelligence in mid-childhood and adolescence in two birth cohorts.^7^^–^^9^ Similarly, in New Zealand, a positive association between cognitive skills and diet was found at 3.5 and 7 years of age.^10^ In Australia, children with healthy diets during early childhood had higher verbal and non-verbal abilities in mid-childhood.^11^ Because diet affects specific outcomes that are important for children’s educational attainment, some authors tested the association of diet with school grades or standardized test scores. A positive association between good diet and academic performance was found in adolescents from Canada,^12^ Chile,^13^ Iceland,^14^^,^^15^ the Netherlands,^16^ Norway,^17^ Sweden^18^^,^^19^ and the United Kingdom.^20^

Research examining how overnutrition affects the academic performance of adolescents has been mainly conducted in high-income countries. However, unhealthy diet in children and adolescents is a major health problem in a growing number of low- and middle-income countries.^21^^,^^22^ Adolescents with good academic performance are less likely to report unhealthy behaviours, including alcohol, tobacco and drug consumption, physical inactivity and risky sex.^23^ Because little is known about the relation between overnutrition and academic performance in adolescents from low- and middle-income countries, we assessed the relation between Chilean students’ diet at the age of 16 years and their academic performance. We hypothesized that a diet in accordance with food guidelines at age 16 years would be associated with better academic performance in both the college admission tests and high school grade-point average.

## Methods

### Study design and population

We studied 16 year-old students living in Santiago, Chile (from a region of low-to-middle socioeconomic status) who were part of a cohort study beginning in infancy. The infants, recruited at 4 months old, were healthy, full-term singletons weighing at least 3 kg at birth. They were assessed for developmental outcomes in infancy, and at age 5, 10 and 16 years. At 16, they were assessed for obesity and cardiovascular risk factors, including body-mass index (BMI), waist circumference, systolic and diastolic blood pressure, blood lipids, glucose and insulin. Enrolment and data from previous waves are described in detail elsewhere.^24^^–^^26^ Data were collected in 2009–2012. Ethical approval was obtained by the institutional review boards of the University of Michigan, Institute of Nutrition and Food Technology (University of Chile) and the University of California, San Diego. Informed consent was provided in writing according to the requirements of the Declaration of Helsinki (1995).

### Dietary assessment

The nutritional quality of diet at age 16 years was measured accounting for the amount of saturated fat, fibre, sugar and salt in the food. We used a validated food frequency questionnaire used in previous studies to assess the usual diet during breakfast, lunch, dinner, snacks at school and snacks at home.^27^ A list of 110 foods and beverages was used. The frequency of food consumption was assessed by a multiple response grid; participants were asked to estimate how often a particular food or beverage was consumed. Categories ranged from never to seven times a week. Software based on the Chilean food composition tables was used to calculate nutrient intake.^28^ Each meal was considered to be: unhealthy (poor nutritional value items, high in fat, sugar, salt and calories); fair (highly processed items although low in fat); or healthy (nutrient rich foods). A score ranging from 0–2 was assigned to each meal category, with higher scores representing healthier habits. To estimate the overall quality of diet, scores were summed as a raw score (range 0–10). We applied cut-offs for the Chilean adolescent population to classify the overall diet of participants into three groups: unhealthy, fair and healthy.^27^

### Academic performance

Academic performance was assessed using the Chilean national standardized exam for college admission. This consists of two mandatory tests (language and mathematics), and two non-mandatory tests (science and social science). Each test is made up of multiple-choice items and scores range from 210–825, with a passing score defined as 450. For this analysis, we considered performance on the mandatory tests only. Scores ≥ 75th percentile in language (521) and mathematics (524) in our sample were defined as good academic performance. Since the exam is just one part of the college application, we also accounted for the academic record as measured by grade-point average (GPA). Following the criteria of the Ministry of Education, grades (on a scale of 1–7) were transformed into scores. The arithmetic average of all annual averages of each subject taken during the four years of high school was calculated and the result was compared in the conversion table provided by the Department of Assessment, Measurement and Educational Record, University of Chile, which compiles specifications and holds the examination on behalf of the Ministry of Education.^29^ Scores ≥ 75th percentile (≥ 566) in our sample were considered good academic performance. We used personal identification codes to link academic results to anthropometric, sociodemographic and dietary data.

### Nutritional status

Standardized procedures were used to measure adolescents’ height and weight. We calculated *Z*-scores for body-mass index (BMI).^30^ Nutritional status was defined as follows: underweight (*Z*-score < −1 standard deviation, SD), normal weight (*Z*-score from −1 SD to 1 SD), overweight (*Z*-score from 1 SD to < 2 SD) and obese (*Z*-score ≥ 2 SD).

### Type of secondary education

In Chile, high school begins after the 8th grade of primary education, lasts for four years and is compulsory. Secondary education includes: academic high school, which provides theoretical education in languages, mathematics, history and sciences; vocational high school, a combination of vocational training and theoretical education; and adult high school, for students who did not achieve a secondary education certificate. Data on the type of secondary education attended by participants was retrieved from the Curriculum and Assessment Unit, Chilean Ministry of Education.

### Maternal education

Because maternal education may affect the quality of the home environment,^31^ this is a potential confounder of the association between diet and academic performance. Maternal education was reported as the highest completed level in three categories: (i) complete elementary education; (ii) complete secondary education; and (iii) complete vocational training or associate degree. We merged these categories into two: incomplete secondary education (i), and complete secondary education or higher (ii and iii).

### Statistical analysis

Statistical analysis included *χ^2^* for categorical variables, Student’s *t*-test and analysis of variance with Bonferroni correction for comparison of means. Associations between diet at age 16 years (the exposure) and academic performance in mathematics, language and GPA (outcomes) were examined using logistic regression models. Potential confounders were selected for inclusion based on statistical significance in bivariate analyses. Four models were estimated. Model 1 included only the nutritional quality of diet as an independent variable. Model 2 also contained the BMI *Z*-score. In model 3, sociodemographic covariates (maternal education and sex) were added to variables in model 1. In model 4 type of secondary education was added to variables in model 3. Odds ratios (OR) are presented in tables with 95% confidence intervals (CI). We evaluated the models using likelihood ratio tests, Hosmer-Lemeshow goodness-of-fit tests and the percentage of correctly classified cases (data available from corresponding author).

## Results

Of 678 adolescents enrolled in the obesity and cardiovascular study, 395 applied for higher education. When comparisons of characteristics were made between those who applied for higher education and those who did not, no differences were found in age, BMI and weight at age 16 years. However, those who did not apply for higher education were less likely to have healthy diets and attend academic high school; a higher proportion of these were males and their mothers were less likely to have completed secondary education (data available from corresponding author).

Our final sample comprised 16.8 (standard deviation, SD: 0.3) year-old males (48%; 189) and females (52%; 206). They completed high school in academic (36%; 142), vocational (54%; 213) and adult schools (10%; 40). Mean scores in language and mathematics were 460.2 (SD: 97.0) and 462.3 (SD: 93.4), respectively, while mean GPA score was 502.4 (SD: 98.2). These scores are similar to those reported for high school graduates in Santiago.

**Fig. 1:**
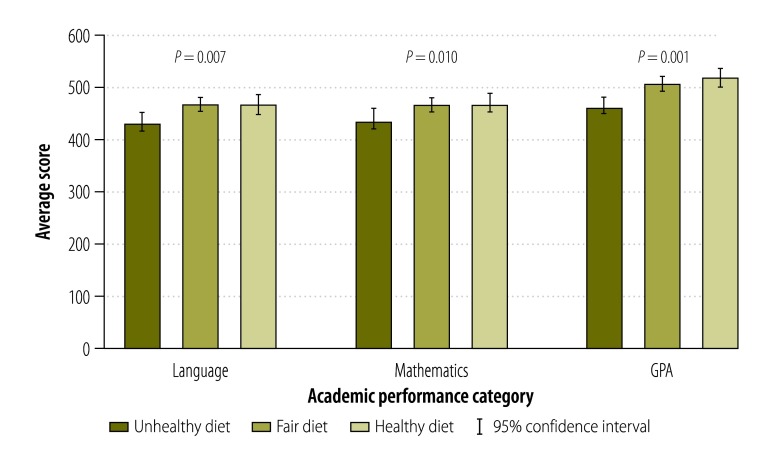
Mean scores in the tests for college admission and high school GPA in Chilean students by nutritional quality of diet, 2009–2012

In this sample, 17% (67) of participants had an unhealthy diet, reporting regular consumption of foods high in fat, sugar, salt and calories. About 50% (197) had fair dietary habits, whereas 33% (131) had a healthy diet (Table 1). Average scores in language, mathematics and GPA increased with higher quality of diet (Table 1 and Fig. 1).

**Table 1:** Characteristics of the study population and nutritional quality of their diet, Chile, 2009–2012

Variable	All (*n* = 395)	Diet category			
		Unhealthy (*n* = 67)	Fair (*n* = 197)	Healthy (*n* = 131)	*P*
**Average age, years**	16.8	16.8	16.8	16.8	NS^a^
**Sex, no. (%)**					
Male	191 (48.4)	27 (40.3)	104 (54.5)	60 (45.8)	NS^b^
Female	204 (51.6)	40 (59.7)	93 (45.5)	71 (54.2)	–
**Anthropometric**					
Average BMI, *Z*-score	0.63	0.78	0.57	0.66	NS^a^
< 1 SD, No. (%)	243 (61.4)	35 (52.2)	131 (66.5)	77 (58.8)	NS^b^
≥ 1 SD, No. (%)	152 (38.6)	32 (47.8)	66 (33.5)	54 (41.2)	–
**Academic performance, average score**					
Language test	460.3	430.6	466.9	465.2	0.007^a^
Mathematics test	462.8	435.3	466.1	467.7	0.010^a^
GPA	502.4	459.7	507.1	515.8	0.001^a^
**Maternal education, no. (%)**					
Incomplete secondary	119 (30.1)	23 (34.3)	56 (28.4)	49 (30.5)	NS^b^
Complete secondary or higher	276 (69.9)	44 (65.7)	56 (71.6)	40 (69.5)	–
**Type of secondary education, no. (%)**					
Academic	142 (36.4)	23 (35.4)	67 (34.1)	52 (40.0)	NS^b^
Vocational	211 (53.9)	32 (49.2)	113 (57.7)	66 (50.8)	–
Adult	38 (9.7)	10 (15.4)	16 (8.2)	12 (9.2)	–

BMI: body mass index; GPA: grade-point average; NS: not significant; SD: standard deviation.

^a^ One-way ANOVA test with Bonferroni adjustment.

^b^ χ^2^ test.

Notes: Academic performance was measured using grade-point average. Diets with poor nutritional value items and high in fat, sugar, salt and calories were classified as unhealthy diets. Diets with highly processed foods that were low in fat were classified as fair diets. Nutrient rich diets were classified as healthy diets.

GPA: grade-point average.

Notes: Diets with poor nutritional value items and high in fat, sugar, salt and calories were classified as unhealthy diets. Diets with highly processed foods that were low in fat were classified as fair diets. Nutrient rich diets were classified as healthy diets. *P*-values show differences between group means and were estimated with analysis of variance with Bonferroni correction. Post hoc analysis was used to identify group means that were significantly different from each other: significantly different from the group with a healthy diet and significantly different from the group with a fair diet.

**Fig. 2:**
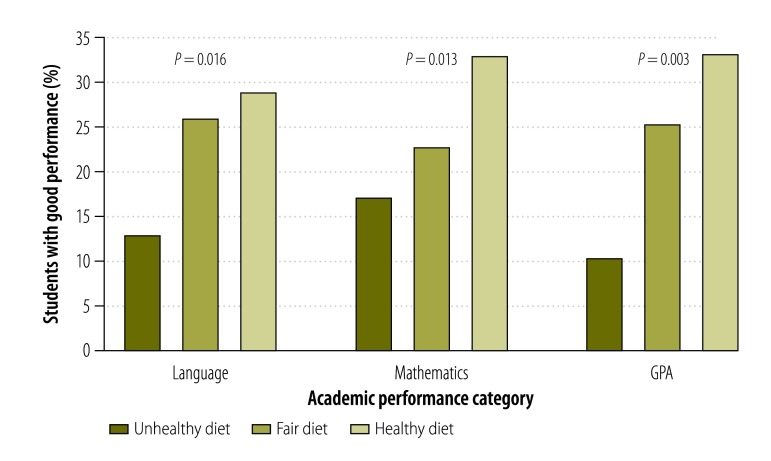
Academic performance and nutritional quality of diet for Chilean students, 2009–2012

Among participants with a healthy diet, the proportion of students with good academic performance was at least twice that in the group with unhealthy dietary habits (Fig. 2). Likewise, in the group of students with good academic performance, there was a higher share of adolescents with healthy diets and a lower share with unhealthy diets compared to students performing below the 75th percentile (results available from corresponding author).

GPA: grade-point average.

Notes: A score ≥ 75th percentile was considered good academic performance. Diets with poor nutritional value items and high in fat, sugar, salt and calories were classified as unhealthy diets. Diets with highly processed foods that were low in fat were classified as fair diets. Nutrient rich diets were classified as healthy diets. *P*-values estimated with Pearson's χ^2^ test.

Compared to participants with healthy diets, those with unhealthy diets were significantly less likely to perform well in language (OR: 0.40; 95% CI: 0.18–0.89) and mathematics (OR: 0.42; 95% CI: 0.19–0.88) (Table 2 and Table 3). Likewise, the odds of having a good GPA were significantly lower among students with an unhealthy diet (OR: 0.26; 95% CI: 0.11–0.61) compared to students with a healthy diet (Table 4). After adjustment for health, sociodemographic and educational confounders (Model 4), the odds of good academic performance did not change substantially and remained significantly lower among students with an unhealthy diet. Also, participants with a fair diet remained less likely to perform well in mathematics (OR: 0.57; 95% CI: 0.32–0.99), compared to those with a healthy diet. Regarding the covariates’ influence, the type of secondary education remained tied to academic performance in the mandatory tests after adjustments, whereas sex remained associated with having a high school GPA (≥ 75th percentile). Maternal education and body weight were no longer significant in models with full adjustment.

**Table 2:** Associations between nutritional quality of diet, aged 16 years, and students’ performance in language tests, Chile, 2009–2012

Variable	OR (95% CI)			
	Model 1	Model 2	Model 3	Model 4
Unhealthy diet	0.40 (0.18–0.89)	0.41 (0.18–0.90)	0.41 (0.18–0.92)	0.42 (0.18–0.98)
Fair diet	0.95 (0.58–1.57)	0.92 (0.56–1.52)	0.92 (0.56–1.52)	0.99 (0.58–1.68)
BMI (*Z*-score)	–	0.78 (0.63–0.96)	0.78 (0.63–0.96)	0.82 (0.65–1.02)
Incomplete maternal education	–	–	0.84 (0.50–1.41)	0.94 (0.61–1.85)
Female sex	–	–	1.07 (0.67–1.71)	0.95 (0.58–1.96)
Vocational high school	–	–	–	0.24 (0.14–0.39)
Adult high school	–	–	–	0.17 (0.06–0.52)

BMI: body mass index; CI: confidence interval; OR: odds ratio.

Notes: Diets with poor nutritional value items and high in fat, sugar, salt and calories were classified as unhealthy diets. Diets with highly processed foods that were low in fat were classified as fair diets. Nutrient rich diets were classified as healthy diets. Reference group: participants with a healthy diet.

**Table 3:** Associations between nutritional quality of diet, aged 16 years, and students’ performance in mathematics tests, Chile, 2009–2012

Variable	OR (95% CI)			
	Model 1	Model 2	Model 3	Model 4
Unhealthy diet	0.42 (0.19–0.88)	0.42 (0.20–0.89)	0.43 (0.20–0.91)	0.35 (0.15–0.82)
Fair diet	0.60 (0.36–0.98)	0.59 (0.36–0.97)	0.58 (0.35–0.93)	0.57 (0.32–0.99)
BMI (Z-score)	–	0.92 (0.75–1.14)	0.94 (0.76–1.15)	0.96 (0.82–1.30)
Incomplete maternal education	–	–	0.61 (0.35–1.04)	0.83 (0.46–1.51)
Female sex	–	–	0.93 (0.58–1.58)	0.75 (0.44–1.25)
Vocational high school	–	–	–	0.14 (0.08–0.25)
Adult high school	–	–	–	0.06 (0.02–0.28)

BMI: body mass index; CI: confidence interval; OR: odds ratio.

Notes: Diets with poor nutritional value items and high in fat, sugar, salt and calories were classified as unhealthy diets. Diets with highly processed foods that were low in fat were classified as fair diets. Nutrient rich diets were classified as healthy diets. Reference group: participants with a healthy diet.

**Table 4:** Associations between nutritional quality of diet, aged 16 years, and students’ academic performance in high school, Chile, 2009–2012

Variable	OR (95% CI)			
	Model 1	Model 2	Model 3	Model 4
Unhealthy diet	0.26 (0.11–0.61)	0.26 (0.11–0.62)	0.24 (0.09–0.58)	0.22 (0.09–0.56)
Fair diet	0.75 (0.46–1.21)	0.73 (0.45–1.19)	0.76 (0.44–1.20)	0.75 (0.46–1.26)
BMI (Z-score)	–	0.82 (0.67–1.01)	0.80 (0.64–1.01)	0.84 (0.68–1.05)
Incomplete maternal education	–	–	0.89 (0.53–1.51)	0.99 (0.58–1.70)
Male sex	–	–	0.46 (0.28–0.74)	0.49 (0.30–0.80)
Vocational high school	–	–	–	0.62 (0.37–1.03)
Adult high school	–	–	–	0.38 (0.14–1.07)

BMI: body mass index; CI: confidence interval; OR: odds ratio.

Notes: Academic performance was measured using grade-point average. Diets with poor nutritional value items and high in fat, sugar, salt and calories were classified as unhealthy diets. Diets with highly processed foods that were low in fat were classified as fair diets. Nutrient rich diets were classified as healthy diets. Reference group: participants with a healthy diet.

## Discussion

The main finding of this study is that Chilean students who had an unhealthy diet at the age of 16 years were less likely to perform well academically. The association between diet and subsequent academic outcomes remained significant after adjusting for BMI, sociodemographic and educational factors.

These findings are relevant to countries undergoing nutritional and epidemiological transitions. While several studies have used self-reported measures of academic performance, we used a nationwide standardized test and school-grades. Our sample is not representative of the Chilean adolescent population; it was made up of adolescents from low-to-middle socioeconomic status. In Chile, an unhealthy diet in people aged 15–24 years was more prevalent among those of low-to-middle socioeconomic status.^32^ Although we accounted for sex, maternal education, BMI and type of secondary education, we did not consider other important influences, such as parental health and marital status; father’s educational level, occupational status and involvement in children’s schooling; the role of peers; or participants’ self-perception. Although previous studies adjusted their results for tobacco and alcohol consumption, we did not include such items in our questionnaire since we felt that many adolescents would not answer truthfully.

Results from studies in other adolescent populations are consistent with ours. In Iceland, performance on language, mathematics and foreign languages was negatively associated with poor diet.^14^^,^^15^ In Norwegian adolescents, a poor diet was significantly associated with performance in mathematics.^17^ Similarly, unhealthy dietary habits at 3, 4 and 7 years were negatively associated with performance on a standardized test administered in the United Kingdom when children were 10–11 years old.^20^ In Chilean students, consumption of energy-dense foods during school snack time was associated with lower odds of success in a nationally standardized test administered to all students aged 9 and 14 years.^13^ In these studies, less healthy foods were defined as those high in processed carbohydrate and/or saturated fat and low in proteins, vitamins and minerals.

Results of studies that have tested the association of academic outcomes with consumption of specific foods are consistent with those using an overall measure of diet. Canadian junior high-schoolers reporting milk, vegetable and fruit consumption on a regular basis had better school-grades.^12^ Daily intake of fruits among Norwegian adolescents was associated with better academic outcomes, independent of nutritional status and parental education.^33^ Swedish students eating fish high in omega-3 fatty acids were more likely to have good school grades.^19^ Finally, in American adolescents, school-grades were inversely associated with daily intake of sugar-sweetened drinks.^34^ These results support recommendations that individuals should consume a diet that meets the requirements of micro and macronutrients needed to support healthy growth and development.^35^

Several dietary components impact on molecular systems or cellular processes that are vital for maintaining cognitive function. In doing so, diet can affect multiple brain processes by regulating neurotransmitter pathways, synaptic transmission, membrane fluidity and signal-transduction pathways.^36^^,^^37^ Omega-3 fatty acids, a key component of neuronal membranes, elevate brain-derived neurotrophic factors, stimulating synaptic plasticity and the efficacy of synaptic transmission.^37^ Flavonoid and non-flavonoid polyphenols, which can be found in fruits and vegetables, modulate learning and memory by promoting neuronal signalling and increasing production of antioxidant and anti-inflammatory agents.^37^ Conversely, excessive exposure to saturated fats and simple sugars decreases levels of hippocampal brain-derived neurotrophic factors and increases oxidative stress.

In our sample of low-to-middle socioeconomic status adolescents, only a third had healthy dietary habits, eating nutrient rich items and protective foods every day. Conversely, 17% ate energy-dense foods, rich in simple sugars and saturated fat and half had only fair dietary habits. Using the same questionnaire we applied in the present study, it has been shown that in the early 2000s, only 13% (220) of 1692 mid-school Chilean students had healthy diets compared to 77% (1303) and 10% (169) who had fair and unhealthy diets, respectively.^27^ Although the share of adolescents having healthy diets increased over the years, there is an increase in the proportion of adolescents with unhealthy diets. Although school health strategies have been developed in Chile since the 1990s, they do not appear to have been successful, due to a lack of coordination among governmental agencies in the implementation of these programmes. The primary aim of these strategies is population health, which is not perceived as a major goal by schools and educational agencies.^38^

These findings have policy implications. Cognitive impairment might contribute to overeating by interfering with learned control of energy regulation.^39^^-^^41^ Evidence that promoting healthy diets in young populations may help prevent cognitive impairment strengthens public health messages about diet based upon physical health alone. Academic performance is closely linked to expectations of parents, school boards and educational agencies and relates to better prospects of jobs, income and socioeconomic status. Many low- and middle-income countries have made an effort to overcome undernutrition and preserve the full cognitive skills of their people. The transition from under- to overnutrition puts this achievement at risk. Future studies should replicate this analysis in other young populations and further investigate how health-related behaviours influence cognitive and academic outcomes.
